# Interest of Widefield-Optical Coherence Tomography Angiography for Diagnosis and Follow-Up of Retinal Neovascularisation in Proliferative Diabetic Retinopathy

**DOI:** 10.1155/2022/5746238

**Published:** 2022-07-31

**Authors:** Brice Nguedia Vofo, Pablo Galarza, Itay Chowers, Jaime Levy

**Affiliations:** Department of Ophthalmology, Hadassah-Hebrew University Medical Center, Jerusalem, Israel

## Abstract

**Aim:**

The primary aim was to evaluate the use of optical coherence tomography angiography (OCTA) versus fluorescein angiography (FA) for detecting and monitoring retinal neovascularization (NV) in patients with proliferative diabetic retinopathy (PDR) receiving treatment with anti-vascular endothelial growth factor (anti-VEGF).

**Methods:**

Treatment-naïve patients with PDR, willing to begin anti-VEGF treatment without laser from 9/2018–2/2020 were included. FA and OCTA scans were obtained at baseline, and a second OCTA scan was performed after 6 months of anti-VEGF therapy. We calculated sensitivity and specificity for two masked graders with respect to identifying NV on OCTA versus FA. Using ImageJ software, we also measured the change in NV size, at baseline and 6-month follow-up.

**Results:**

Ten eyes in eight patients were included, of which three eyes in three patients received a 6-month follow-up examination. Mean age was 51.7 ± 11.2 years, and 75% of patients were male. Overall, 21 NV sites in the 10 eyes were identified both clinically and on FA. Using OCTA scans, the sensitivity and specificity for both graders were extremely high, ranging from 95.2% to 100%. At 6-month follow-up, NV size decreased by 69.8%.

**Conclusion:**

These results suggest that OCTA may provide a suitable alternative to FA for visualizing, measuring, and monitoring changes in retinal NV in patients with PDR who receive anti-VEGF therapy.

## 1. Background

Two relatively recent prospective randomized clinical trials examined the treatment efficacy of intravitreal injections of anti-vascular endothelial growth factor (anti-VEGF) in patients with proliferative diabetic retinopathy (PDR) [1, 2]. Both trials examined patients every 4 weeks, including a fundus examination and either seven-field or widefield fundus photography, to monitor the regression or recurrence of retinal neovascularization (NV). However, these techniques can have limitations with respect to detecting the early stages of newly developed vessels [3]. The current gold standard for detecting retinal NV is fluorescein angiography (FA); however, performing FA every 4 weeks to monitor NV growth or regression and ischemic retinal areas is contraindicated due to the need for repeated injections of fluorescein dye.

On the other hand, few prospective studies evaluated the effects of anti-VEGF injections in PDR cases using widefield optical coherence tomography angiography (OCTA), even though this technique can detect retinal NV with high accuracy [4]. The ability to monitor these clinical parameters using OCTA would provide valuable insights into the effects of anti-VEGF therapy on the retinal vasculature and patients' response to treatment.

Here, we used widefield OCTA to prospectively monitor the retinal neovascularization in treatment-naïve PDR cases at baseline and after 6 months of intravitreal anti-VEGF therapy.

## 2. Methods

### 2.1. Study Design

In this prospective study, consecutive subjects were recruited from the retina clinic at the Hadassah-Hebrew University Medical Center in Jerusalem, Israel, from September 2018 through February 2020. Each participating subject provided written informed consent obtained in accordance with the Declaration of Helsinki and the Hadassah Ethics Committee. Patients with treatment-naïve eyes diagnosed with PDR in which anti-VEGF therapy was indicated were eligible for inclusion. Additional inclusion criteria included good media clarity and a good view of the fundus for imaging. The Diabetic Retinopathy Severity Scale (DRSS) score was measured in all cases. Exclusion criteria included pregnancy, laser photocoagulation, and/or intraocular surgery performed within the previous 3 months, intraocular inflammation, myopia >8 diopters, and any other condition that might affect the retinal vasculature. We also excluded patients with known allergies and those who were medically contraindicated to receive fluorescein dye injections. For each patient included in the study, PDR with retinal NV was identified by clinical examination, and leakage was confirmed with FA.

### 2.2. Data Collection

The biometrics and baseline characteristics of eligible patients (age, gender, and past ocular and medical history) were recorded. The patients underwent a baseline ophthalmic examination, including Early Treatment Diabetic Retinopathy Study (ETDRS) visual acuity, biomicroscopy, spectral-domain OCT (SD-OCTA; Heidelberg Engineering, Heidelberg, Germany), widefield fundus photography, FA using an Optos 200Tx ultra-widefield imaging device (Optos, Dunfermline, UK), and widefield swept-source OCTA using a Zeiss PLEX Elite 9000. To capture as much of the periphery as possible, we obtained 12 mm × 12 mm scans (including both OCTA cross-sectional and angiography scans) and then used the montage function of the Advanced Retinal Imaging (ARI) network image analysis software. To maximize repeatability in the follow-up scans, we used the automated eye-tracking option when performing the OCTA scans. OCTA scans with low quality due to movement artifacts, unstable fixation, and/or or poor focusing were systematically excluded, and new scans were obtained until suitable quality scans were achieved. We also manually cross-checked and corrected any errors due to automated segmentation. Using montage images enabled us to view an en face image of all retinal layers, including the vitreo-retinal interface.

### 2.3. Patient and Public Involvement

Patients were not involved in the design or conduct of the study, the choice of outcome measures, or the recruitment of participants. The patients will not be involved in disseminating the results.

### 2.4. Measurements and Statistics

Sensitivity and specificity of identifying retinal NV between OCTA, FA, and pseudocolor fundus photographs were compared. The change between baseline and the 6-month follow-up visit in area of retinal neovascularization at the optic disc (NVD) and elsewhere (NVE) was measured.

Retinal NV was graded by two independent board-certified ophthalmologists who were trained by the company's representatives to use the OCTA PLEX Elite 9000, and they were masked with respect to the patients' fundus pictures and FA scans. The graders were instructed to indicate what they perceived as NV sites on the en face images of the OCTA scans (vitro-retinal interface, superficial retinal, and whole retina slabs), and 1 week later, they were again instructed to indicate what they perceived as NV sites on the en face and cross-sectional OCTA images. The areas indicated as NV sites on OCTA by the two graders were noted and compared to the sites detected in the clinic and using FA. The sensitivity and specificity of OCTA for detecting NV were estimated and compared to the results obtained on FA, which is currently considered the gold standard.

Patients who opted to receive anti-VEGF treatment alone received three monthly intravitreal anti-VEGF injections, followed by injections administered PRN (*pro re nata*) based on the assessment of retinal NV on ophthalmoscopy and widefield fundus photography in accordance with the Diabetic Retinopathy Clinical Research Network (DRCR.net) Protocol S. Anti-VEGF injections were administered in the outpatient ophthalmology clinic at Hadassah Medical Center as part of the regular treatment given to patients with PDR.

Using the freehand drawing tool in ImageJ, we delineated and measured the size of each retinal NV at baseline and, where applicable, 6 months after the start of anti-VEGF injections.

Statistical analyses were performed using SPSS version 25.0 (IBM Corp., Armonk, NY). Frequency counts and percentages were generated where appropriate.

## 3. Results

A total of 10 patients initially enrolled in the study; however, two patients were subsequently excluded when good images could not be obtained in the study eye due to poor fixation. We therefore analyzed 10 eyes in 8 patients. The baseline characteristics of these 8 patients are summarized in Table 1. According to the DRSS score, six eyes were graded as level 61, while 4 eyes were graded as level 65. We then prospectively evaluated three eyes (two with NVE and one with NVD) in three patients who received anti-VEGF injections alone. All three patients had no diabetic macular edema (DME) and all received a total of six monthly injections of bevacizumab over a 6-month period. None of these patients developed vitreous hemorrhage.

**Figure 1:**
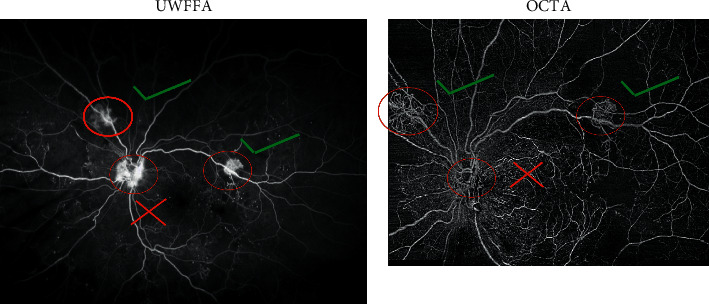
Example of a retinal neovascularization (NV) site that showed leakage on the ultra-widefield fluorescein angiography (UWF-FA) scan (left) but was missed by one of the graders on the en face optical coherence tomography angiography (OCTA) scan (right). A total of three NV sites are present (red ovals); the two with the green check-mark were identified on both scans, while the site with the red “X” was not seen on en face OCTA images.

**Figure 2:**
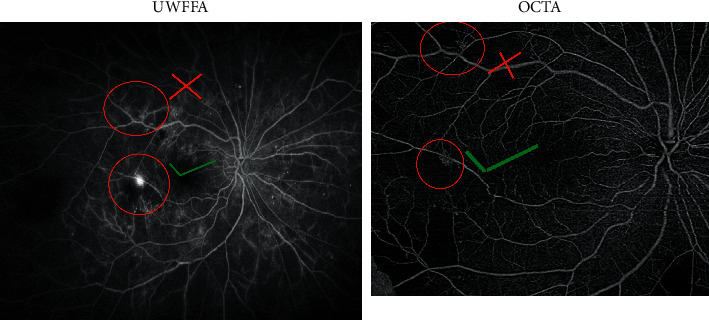
Example of a retinal NV site incorrectly identified (i.e., a false positive) on en face OCTA by one of the graders. Only one of the two NV sites identified on OCTA (the site with the green check-mark) was confirmed on the UWF-FA scan; the other site (with the red “X”) was not confirmed on UWF-FA.

**Figure 3:**
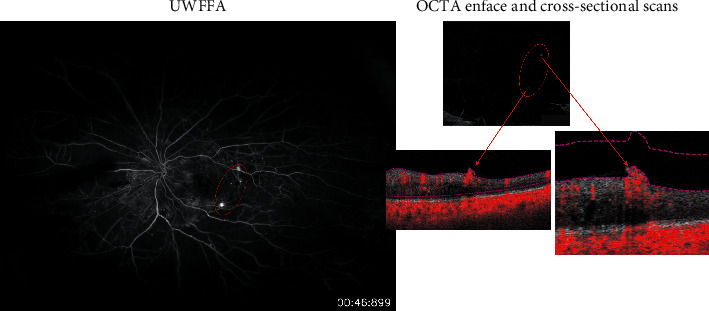
Example of two retinal NV sites seen on UWF-FA (left) and identified on both the en face OCTA scan (top right image) and the corresponding cross-sectional OCTA scans (bottom right images) as extending above the vitreo-retinal interface.

At baseline, a total of 21 NV sites were identified clinically in the 10 study eyes and were confirmed as leakage sites on FA. The sensitivity and specificity of both graders for identifying retinal NV using the en face images alone and using both the en face images and cross-sectional scans are summarized in Table 2. Sensitivity was defined as the number of retinal NV sites identified on OCTA divided by the number of sites confirmed on FA. To determine specificity, each area incorrectly identified as an area of retinal NV counted as a negative point out of a preset total of 21points, corresponding to the total number of NV sites. Using the en face OCTA images alone, the identification of retinal NV was less sensitive and less specific compared to using a combination of both en face cross-sectional OCTA images.  Figure 1 shows an example of a NV site that was seen clinically and confirmed to be leaking on FA but was missed (false negative) by one of the graders when given only the en face image; in contrast, Figure 2 shows an example of a site that was incorrectly labeled as a NV site by one of the graders but was not identified as NV either clinically or on FA (false positive). Moreover, Figure 3 shows an example of two NV sites identified on a cross-sectional OCTA scan and confirmed on FA.

**Figure 4:**
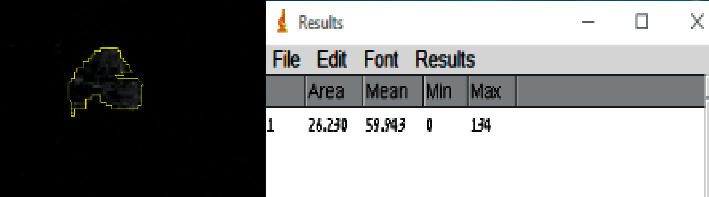
Example of the method used for the demarcation (left, yellow outline) and measurement of retinal NV using the OCTA scan and ImageJ (right).

The size of the NV sites in the three eyes that received anti-VEGF injections, measured as shown in Figure 4, is summarized in Table 3. Relative to baseline, the size of the NV sites decreased by an average of 69.8%.

## 4. Discussion

In this study, we compared the ability to identify newly formed retinal vessels between OCTA and ultra-widefield FA (UWF-FA) scans; specifically, we compared this difference when using only en face OCTA images and when using both the en face and cross-sectional OCTA scans. We found that when given the en face images and the ability to scroll through the cross-sectional OCTA scans, both graders achieved a high level of precision with respect to accurately identifying retinal neovascularization, comparable to the precision obtained using the current gold standard of FA.

These results are consistent with the recent report by Sawada et al., who found high sensitivity and specificity with using both en face and cross-sectional OCTA scans compared to UWF-FA scans [4]. Interestingly, we found that both graders in our study were less accurate at correctly identifying retinal NV when given only the en face images. Specifically, the graders tended to miss NV sites located relatively close to the optic nerve head and NV sites that arose from areas that did not have a low surrounding capillary density. Using the en face OCTA images alone, both graders also tended to mislabel areas of intraretinal microvascular abnormalities (IRMAs) as sites of neovascularization. In contrast, when given both the en face and cross-sectional images, both graders easily delineated the NV as extending above the vitreo-retinal interface, providing extremely high sensitivity and specificity for detecting retinal NV. It is important to note that all of the automated segmentation was manually crossed-checked and corrected as needed before the scans were presented to the graders. Recently, Hirano et al. reported that manually correcting automatically segmented OCTA scans increased the sensitivity for detecting retinal NV from 73% to 84%, as an NV lying flat on the internal limiting membrane surface can easily be missed due to segmentation errors [5].

As the use of OCTA increases worldwide, several novel OCTA biomarkers have now been identified as possible tools to better access and monitor patients with diabetic retinopathy [6–9]. Previous studies such as the Diabetic Retinopathy Clinical Research Network (DRCRN) Protocol S study [1] and the CLARITY study [2] revealed putative advantages of treating PDR with anti-VEGF injections versus pan-retinal laser photocoagulation. In the DRCRN study, the Diabetic Retinopathy Severity Scale (DRSS) scores—which they graded primarily using color fundus photographs—improved by approximately 2 stages, reflecting regression of the retinal NV, microaneurysms, and IRMAs. However, using measurements of NV size on OCTA, we provide the first prospective quantitative measurements of the actual percent change in retinal NV size after treatment with anti-VEGF injections for 6 months, with an overall reduction of 69.8% compared to baseline. These quantitative results are consistent with the qualitative results reported by the CLARITY study, in which the retinal NV had fully regressed by 52 weeks in the majority of patients [2]. Furthermore, Lupidi et al., in a prospective evaluation of vascular perfusion densities and retinal NV 1 month after PRP treatment, hypothesized that a reduction of 40% in retinal neovascular size could be a biomarker of “laser efficacy.” This is after they observed a 41% mean reduction in the retinal NV size in patients that did not need retreatment, compared to 20% in those who needed retreatment. The 69.8% reduction in retinal NV seen after 6 months in our patients could therefore be indicative of a successful treatment [9].

Our study has limitations that warrant discussion, including the relatively small sample size. We had only 3 eyes in prospective arm evaluating change in neovascularization size and did not account for confounders like diabetic control over the 6 months of follow-up. These results should therefore be verified in larger prospective studies. In addition, our use of the freehand drawing tool in ImageJ to delineate and measure NV size could have led to a certain degree of inaccuracy due to the challenges associated with perfectly delineating the irregular edges of an NV site. To minimize this possibility, we enlarged the images as much as possible in ImageJ to improve visualization and demarcation of the NV edges. We also used relatively large OCTA scans (12 mm × 12 mm) with montage images, producing widefield images to match the UWF-FA images and reducing the risk of missing an NV site in the periphery, as previously described [10]. Finally, we identified a few segmentation errors made by the automated software; however, these errors were corrected manually in order to prevent inaccurate measurements.

In conclusion, our findings suggest that using a combination of en face and cross-sectional OCTA images provides extremely high sensitivity and specificity for detecting retinal NV, compared to the results obtained using the current gold standard, fluorescein angiography. We also report that OCTA scans can be used to demonstrate a decrease in NV size in eyes treated for 6 months with anti-VEGF injections.

## Figures and Tables

**Table 1:** Baseline characteristics of study participants (*N* = 10 eyes in 8 patients).

Patient characteristics	*N* (%) or mean ± SD
Gender	
Female	2 (25%)
Male	6 (75%)
Age	51.7 ± 11.2
HbA1c	9.2 ± 1.3%
Laterality	
Right eye	5 (50%)
Left eye	5 (50%)
LogMAR BCVA	0.14 ± 0.16
Pseudophakia	
Yes	2 (20%)
No	8 (80%)
Type of diabetes	
Type 1	0 (0%)
Type 2	8 (100%)
Hypertension	
Yes	3 (37.5%)
No	5 (62.5%)
Ischemic heart disease	
Yes	1 (12.5%)
No	7 (87.5%)

HbA1c, hemoglobin A1C; LogMAR, logarithm of the minimum angle of resolution; BCVA, best-corrected visual acuity.

**Table 2:** Sensitivity and specificity of two masked graders in the detection of retinal NV on OCTA relative to UWF-FA.

	Grader 1 (%)	Grader 2 (%)
*En face OCTA alone*		
Sensitivity	85.7	81.0
Specificity	90.5	95.2

*En face OCTA plus cross-sectional OCTA*		
Sensitivity	100	100
Specificity	100	95.2

NV, neovascularization; OCTA, optical coherence tomography angiography; UWF-FA, ultra-widefield fluorescein angiography.

**Table 3:** Summary of retinal neovascularization size at baseline and at the 6-month follow-up.

Case no.	Area at baseline (*μ*m^2^)	Area at 6 months (*μ*m^2^)	Change (*μ*m^2^)
Case 1	102.353	26.891	−75.462
Case 2	81.254	28.298	−52.956
Case 3	26.230	8.121	−18.109

## Data Availability

The datasets used and/or analyzed during the current study are available from the corresponding author on reasonable request.
